# Pyruvate Kinase M2: A Potential Target for Regulating Inflammation

**DOI:** 10.3389/fimmu.2016.00145

**Published:** 2016-04-21

**Authors:** Jose C. Alves-Filho, Eva M. Pålsson-McDermott

**Affiliations:** ^1^Department of Pharmacology, Ribeirao Preto Medical School, University of São Paulo, Ribeirao Preto, Brazil; ^2^Biomedical Science Institute, School of Biochemistry and Immunology, Trinity College Dublin, Dublin, Ireland

**Keywords:** PKM2, inflammation, HIF-1α, glycolysis and oxidative phosphorylation, immunometabolism, cancer

## Abstract

Pyruvate kinase (PK) is the enzyme responsible for catalyzing the last step of glycolysis. Of the four PK isoforms expressed in mammalian cells, PKM2 has generated the most interest due to its impact on changes in cellular metabolism observed in cancer as well as in activated immune cells. As our understanding of dysregulated metabolism in cancer develops, and in light of the growing field of immunometabolism, intense efforts are in place to define the mechanism by which PKM2 regulates the metabolic profile of cancer as well as of immune cells. The enzymatic activity of PKM2 is heavily regulated by endogenous allosteric effectors as well as by intracellular signaling pathways, affecting both the enzymatic activity of PKM2 as a PK and the regulation of the recently described non-canonical nuclear functions of PKM2. We here review the current literature on PKM2 and its regulation, and discuss the potential for this protein as a therapeutic target in inflammatory disorders.

## Introduction

Cancer cells and most activated immune cells display a radical shift in metabolism becoming highly dependent on glucose, which is metabolized through an increased rate of aerobic glycolysis, a metabolic state termed the Warburg effect (1, 2). Normal cell metabolism involves generating energy through a relatively low rate of glycolysis giving rise to pyruvate, which enters the mitochondrial tricarboxylic acid (TCA) cycle. Pyruvate undergoes a series of oxidizing reactions, thereby generating ATP. In contrast, cells displaying Warburg metabolism will instead rely on an increased rate of glycolysis to generate energy. Pyruvate is now diverted away from the oxidative phosphorylation of the TCA cycle and is converted to lactate by lactate dehydrogenase (LDH) in the cytosol. Since this process allows for ATP generation during low oxygen, it may provide an explanation for the tolerance of cancer cells to extreme local hypoxia providing the cells with obvious growth advantages compared to surrounding tissue and immune cells. The high rate of glycolysis ensures that the increased demand for biosynthetic precursors, including proteins, lipids, and nucleic acids, is met. As glucose is broken down to pyruvate, intermediates of glycolysis are used for nucleotide and amino acid synthesis as well as for nicotinamide adenine dinucleotide phosphate (NADPH) production through the pentose phosphate pathway (PPP). Furthermore, fatty acids, required for membrane lipid synthesis, are synthesized from citrate in the cytosol generating acetyl-CoA. This metabolic reprograming renders the cells highly dependent on glucose, which can lead to nutrient competition within the tumor microenvironment, a scenario that has been shown to directly contribute to cancer progression (3). Interest in the metabolic state of immune cells during inflammation and infection has recently surged as it is becoming clear that resting immune cells display distinct metabolic configurations compared to activated immune cells. Hence, the field of immunometabolism has evolved incorporating the concept that alterations in metabolism may influence the phenotype of immune cells and regulate transcriptional, as well as posttranscriptional events, upon activation.

Pyruvate kinase (PK) is the enzyme responsible for the final rate-limiting step of glycolysis, catalyzing phosphoenolpyruvic acid (PEP) and ADP to pyruvate and ATP. Due to the vast literature supporting the role of the PK isoform PKM2 as a key regulator of the metabolic changes observed in cancers [reviewed recently in Ref. (4, 5)], an interest in defining the potential role of this protein in inflammation has emerged. Here, we will review our understanding of PKM2’s regulation and functions in cancer and immune cells, and examine the current literature on its role in inflammatory disorders while discussing the potential in targeting PKM2 function therapeutically.

## PKM2 Gene Expression

Pyruvate kinase isozyme type M2 (PKM2) is one of the four PK isoforms expressed in mammalian cells and is generally accepted to be the embryonic isoform, also expressed in cancer and normal proliferating cells such as lymphocytes and intestinal epithelial cells (6–8). PKM1 is the alternatively spliced product of the same *Pkm* gene (9–11). PKM1 has high PK enzymatic activity and is expressed in tissues with increased catabolic demands such as heart, muscle, and brain. The remaining isoforms PKL and PKR are expressed in the liver and red blood cells, respectively.

PKM1 and PKM2 are generated by exclusive alternative splicing of a pair of mutually exclusive exons of the *Pkm* pre-mRNA. The full open reading frame is composed of 12 exons where inclusion of exon 9 will generate PKM1 transcript, and exon 10 is specific for expression of PKM2 (9, 11). Although only different by a small number of amino acids, the two gene products display distinct function and characteristics due to the isoform specific exons giving rise to structural differences in the fructose-1,6-bisphosphate (FBP)-binding site (discussed below) and dimer–dimer interface.

Two regulatory events have been identified resulting in reciprocal effects on the mutually exclusive exons 9 and 10, such that exon 9 is repressed and exon 10 is activated. First, three heterogeneous nuclear ribonucleoproteins (hnRNPs) polypyrimidine tract-binding protein (PTB, also known as hnRNPI), hnRNPA1 and hnRNPA2, have been shown to bind specifically and repressively to sequences flanking exon 9 resulting in exon 10 inclusion (12). These hnRNP proteins are in turn controlled by c-Myc, contributing to deregulated PK mRNA splicing in cancer. Second, the serine/arginine-rich splicing factor 3 (SRSF3) will, through binding within exon 10, promote its inclusion, resulting in increased transcript for PKM2 (13).

Evidence supports a switch in the expression of PKM1 in favor of PKM2 during malignant transformation such that expression of PKM1 decreases proportionally as the expression of PKM2 increases. However, this has recently been reevaluated, suggesting that upregulation of PKM2 is primarily due to the elevated transcriptional levels of the entire *Pkm* gene, where no decrease in PKM1 expression is observed, rather than due to a switch in isoform expression (7, 14, 15).

In addition, efforts to identify specific micro-RNAs (miRs) that target PKM2 expression have revealed a possible role for miR-let-7a, miR-122, miR-326, miR-133a, and miR133b (16–19); however further validation will be required.

## Regulation of PKM2 Activity

Since PKM2 plays a critical role in the metabolic changes observed in cancer and inflammation, discovering the mechanism of the regulation of PKM2 activity is important to our understanding of how alterations in cellular metabolism are controlled.

The enzymatic activity of PK is, in part, determined by the configuration of the enzyme into a tetramer, dimer, or monomer. PKM1 naturally exists as a stable tetramer, which allows for optimal binding of the substrate PEP. Experiments using partially denatured PKM1 demonstrate that the monomeric and dimeric forms retain only a fraction of the PK activity observed with PKM1 as a tetramer (20).

**Figure 1:**
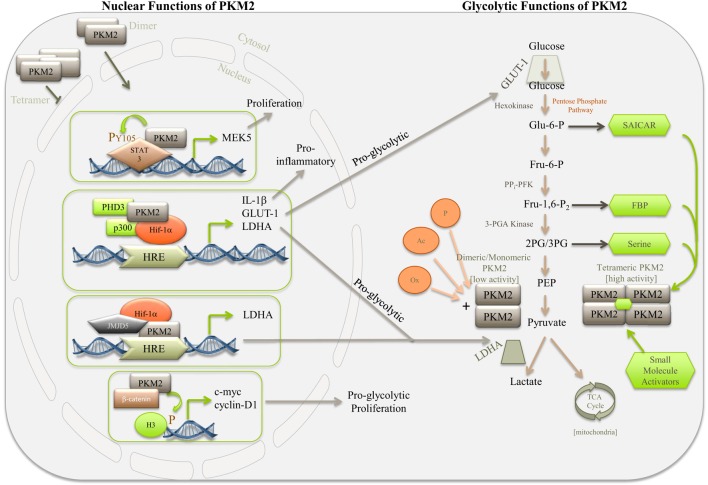
**Simplified diagram depicting some of the nuclear and glycolytic functions and regulation of PKM2**. PKM2 is the major PK isoform expressed in cancer, proliferating cells, and populations of activated immune cells. The activity of PKM2 can be controlled by stabilizing or destabilizing the formation of PKM2 tetramers. Allosteric activation of PKM2 by, for example, succinylaminoimidazolecarboxamide ribose-5′-phosphate (SAICAR), serine, FBP, or small-molecule activators, such as TEPP 46 and DASA 58, encourages tetramer formation of PKM2, thereby promoting the last rate-limiting step of glycolysis, converting PEP to pyruvate. Pyruvate will enter the TCA cycle of the mitochondria where it is used to generate ATP through oxidative phosphorylation. In the absence of allosteric activators PKM2 primarily takes on a dimeric or monomeric form, which through lacking enzymatic activity will give rise to accumulation of glycolytic intermediates, thereby meeting the requirements for biosynthetic precursors of the activated or proliferating cell. Dimeric PKM2 can translocate to the nucleus where it further promotes aerobic glycolysis through Hif-1α co-activation, aiding expression of proglycolytic genes, such as *ldha* and *glut-1*, as well as proinflammatory IL-1β. Furthermore, PKM2 can interact with other transcription factors, such as STAT3, as well as histone H3 and JMJD5, thereby further regulating genes important for proliferation and glycolysis.

On the other hand, PKM2 requires binding of an activator in order to trigger high enzymatic PK activity (Figure 1). PKM2 can be allosterically activated by multiple endogenous regulators that affect binding affinity of PEP to the active site on the enzyme. One such example is FBP, an upstream glycolytic intermediate (21). In the absence of FBP, PKM2 even as a tetramer has a low affinity for PEP. Binding of FBP to PKM2, at a site distinct to the active PEP binding site, will promote and stabilize tetramer formation of PKM2 as well as increase PEP binding affinity, making the kinetic parameters of PKM2 almost identical to those of PKM1.

In addition to FBP, other non-glycolytic metabolites, amino acids, and small molecules also affect PKM2 activity. The small-molecules DASA 58 and TEPP 46 are highly specific activators of PKM2 (22–24). They bind to PKM2, at a site distinct from the FBP binding site, resulting in PKM2 forming a tight tetramer with PKM1-like kinetic properties, an event that is resistant to inhibition by tyrosine phosphorylation (see below). In cancer cells, as well as in activated macrophages, the increase in PKM2 expression and the decrease in overall PK activity will allow for glycolytic intermediates to be channeled into production of, for example, serine and glycine (23). This increased metabolic flux into serine and glycine biosynthetic pathways is critical for cancer cell survival [for review, see Ref. (25)]. It is, therefore, not surprising that a link between serine abundance and PKM2 activity has been reported, where serine is shown to act as a natural ligand and allosteric activator of PKM2 (26) (Figure 1). In a similar manner, cellular accumulation of the *de novo* purine nucleotide synthesis intermediate SAICAR promotes cancer cell survival through interaction of SAICAR with PKM2 (27). Since SAICAR is synthesized as a by-product of glutaminolysis and can be cleaved to provide the TCA cycle with fumarate, this interaction allows for a potential mechanism to convey cellular metabolic demands to PKM2.

Death-associated protein kinase (DAPk) is a serine/threonine kinase with tumor suppressor properties that was identified as binding to PKM2 in a yeast-two-hybrid screen (28). The direct binding of DAPk to PKM2 increases the PK activity of PKM2 and provides another means of regulating the cellular glycolytic rate.

PKM2 enzymatic activity can also be allosterically inhibited. Binding of phenylalanine to a site distinct from both the active site and the FBP binding site will decrease the affinity of PEP to PKM2 through stabilizing PKM2 in an inactive tetrameric form (29, 30). Alternatively, the same site can be occupied by alanine, a scenario that promotes dissociation of PKM2 into a less-active dimeric form. Moreover, the thyroid hormone triiodo-l-thyronine (T3) stabilizes an inactive monomeric form of PKM2, an inhibitory event that can be overcome by binding of PKM2 to FBP (31, 32). Furthermore, tyrosine phosphorylation has been reported as a mechanism for negatively regulating PKM2, thereby promoting tumor growth (33). This phosphorylation event on tyrosine 105 (Y105) disrupts tetramer formation of PKM2 by releasing FBP, thereby regulating the switch from oxidative phosphorylation to aerobic glycolysis. In addition, Y105 phosphorylation of PKM2 by nucleophosmin–anaplastic lymphoma kinase (NPM–ALK) results in decreased enzymatic activity of PKM2 in anaplasic large-cell lymphoma, supporting a role for NPM–ALK in the regulation of metabolism (34). Another kinase important in regulating PKM2 activity in hepatocellular carcinoma is the proapoptotic enzyme JNK-1, which phosphorylates PKM2 at threonine 365. JNK-1 activity in turn is negatively regulated by poly(ADP-ribose) polymerase (PARP)14, which thereby regulates Warburg metabolism, and promotes cell survival and tumor growth (35). A role for *O*-GlcNAc transferase regulating serine phosphorylation and *O*-GlcNAcylation levels of PKM2 in colorectal cancer has also been reported (36).

Shikonin and its derivatives are also inhibitors of PKM2 activity (37, 38). Shikonin is a naturally occurring naphthoquinone isolated from the herb *Lithospermum erythrorhizon* and has been investigated as a potential anticancer drug. Shikonin and its analog alkannin showed promising selectivity toward PKM2, since they did not inhibit PKM1 and PKL activity at IC_50_ to PKM2 (38).

Superfluous production of reactive oxygen species (ROS), commonly associated with cancer cells, requires detoxification by the tripeptide glutathione (GSH). GSH in turn is maintained in the cell by the reduced form of NADPH, which is provided by the PPP. This increase in intracellular ROS has been shown to be alleviated by the inhibition of PKM2 through oxidation of Cys^358^ (39). This inhibitory event will promote glucose flux into the PPP, providing the reducing power required for ROS detoxification and promoting cancer cell survival during conditions of oxidative stress.

## Non-Glycolytic Processes

In addition to being an important control point in glycolysis, PKM2, upon mitogenic, oncogenic, and LPS stimulation, also translocates to the nucleus where it regulates the expression of numerous proglycolytic enzymes (Figure 1). In cells activated with EGRF, ERK2 binds directly to PKM2 and phosphorylates Ser37 on PKM2, leading to recruitment of peptidyl-prolyl *cis*–*trans* isomerase NIMA-interacting 1 (PIN1). PIN1 aids binding of PKM2 to importin α5, thereby facilitating translocation of PKM2 to the nucleus (40, 41). Modification of PKM2 through sumoylation by the SUMO–E3 ligase as well as acetylation by p300 acetyltransferase will prevent binding of PKM2 to FBP and promote nuclear translocation (42, 43). A recent study proposes a role for sirtuin 6 (Sirt6) in regulating nuclear localization of PKM2. Sirt6 will bind and deacetylate PKM2 at lysine 433, thereby promoting nuclear export resulting in reduced cell proliferation and oncogenic properties of PKM2 (44). Furthermore, enhanced tetramer formation of PKM2 using TEPP 46 and DASA 58 will prevent nuclear localization of PKM2 (23, 45). In addition, nuclear PKM2 has been linked to caspase-independent programed cell death (46).

In cancer cells, PKM2 has been shown to function as a coactivator of hypoxia-inducible factor 1-alpha (HIF-1α) (Figure 1). HIF-1α is a key mediator of the Warburg effect and was originally identified as part of a family of transcription factors responsive under conditions of low oxygen or hypoxia. HIF-1α plays a critical role in the induction and maintenance of aerobic glycolysis, partly through inducing expression of glycolytic enzymes. Prolyl hydroxylase 3 (PHD3) acts as a cofactor to PKM2, promoting HIF-1α transactivation of target genes including lactate dehydrogenase (LDH), the glucose transporter GLUT-1, and pyruvate dehydrogenase kinase-1 (PDK-1) (47, 48).

Expression of Jumonji C domain-containing dioxygenase 5 (JMJD5) has been linked to carcinogenesis and regulates PKM2 activity by binding and preventing PKM2 tetramers to form, thereby blocking the enzymatic activity and promoting nuclear translocation (45). PKM2 together with HIF-1α and JMJD5 are then recruited to the HRE element of LDHA (Figure 1). PKM2 can also bind and regulate the activity of octamer-binding transcription factor 4 (Oct-4), a protein important for the maintenance and regulation of undifferentiated stem cells (49).

Recent findings propose a role for nuclear PKM2 as a transcriptional coactivator of c-Src-phosphorylated β-catenin as well as in promoting phosphorylation of histone H3 by PKM2 in EGFR-activated cells (50, 51) (Figure 1). Numerous other reports have confirmed the protein kinase function of PKM2, where PKM2 catalyzes transfer of phosphate from PEP to serine, threonine, or tyrosine residues on target substrates. Phosphorylation of histone H3 suggests a critical role for PKM2 in the epigenetic regulation of gene transcription in the metabolic switch observed during Warburg metabolism, as well as in G1–S phase transition of the cell cycle (51). Furthermore, PKM2 may also regulate the cell cycle through phosphorylation of important cell cycle regulators, including Bub3 and myosin light chain 2 (MLC2), to initiate cytokinesis (52). Nuclear PKM2 directly phosphorylates STAT3 on tyrosine 107-promoting transcription of MEK-5 (53). However, recent data failed to demonstrate PKM2-dependent phosphorylation *in vitro* using either PEP or ATP as phosphate donors, questioning the role of PKM2 as a protein kinase (54).

## PKM2 as a New Player in Inflammation

Understanding the intricate interplay between cell signaling and metabolic pathways has emerged as an important focus of research in the field of cancer and, most recently, in inflammation.

Inflammation is a well-controlled process triggered by signals from damaged tissue or infection aiming to re-establish tissue homeostasis. It is a complex reaction that starts with activation of the “*front-line*” resident leukocytes (i.e., macrophages and dendritic cells) that leads to activation of surrounding microcirculation, and recruitment of neutrophils and other leukocytes to infected/damaged foci (55). Therefore, the inflammatory response is an energy-intensive process that involves a dramatic switch from a resting to a highly active metabolic state. This metabolic reprograming thereby directs nutrients to the efficient generation of ATP and synthesis of macromolecules that are required for the production of proinflammatory mediators, cytoskeleton rearrangement, and proliferation by immune cells. In this realm, it is not surprising that such highly active inflammatory cells undergo a metabolic shift from oxidative phosphorylation to aerobic glycolysis, resembling the well-described Warburg effect found in tumor cells. Indeed, it is becoming increasingly clear that metabolic enzymes and their regulators, initially implicated in the control of cellular metabolism, also display critical roles in regulating immune cell functions. Thus, immune cell metabolism has become a new attractive target area for the development of potential therapies for inflammatory diseases.

Although the full picture in cancer progression still needs to be resolved, increased expression of PKM2 has been reported in a wide range of tumors. Accumulating evidence suggests a central role of this protein in regulating the Warburg effect and many biological processes in cancer cells, including proliferation and survival [for review, see Ref. (56, 57)]. Emerging evidence has also implicated PKM2 as critical regulator of immune cell metabolism and functions *via* regulating the Warburg effect, supporting its potential role in the genesis of inflammation. It has been shown that the expression of PKM2 is strongly increased in LPS-activated macrophages, mainly in a less-active monomeric/dimeric conformation and phosphorylated state (23, 58, 59). As mentioned above, the less active monomeric/dimeric form of PKM2 drives aerobic glycolysis, while the active PKM2 tetramer provides pyruvate for the TCA cycle. Thus, the expression PKM2 in LPS-activated macrophages adds another piece to the puzzle of metabolic reprograming toward aerobic glycolysis in activated macrophages. Meanwhile, LPS-induced PKM2 translocates into the nucleus and forms a transcriptional complex with HIF-1α that directly binds to the IL-1β promoter gene and activates its transcription. This highlights the interplay between metabolic reprograming and control of gene expression in activated macrophages induced by PKM2. Driving PKM2 into tetramer conformation with DASA-58 and TEPP-46 inhibited LPS-induced nuclear translocation and, subsequent LPS-induced expression of IL-1β and a range of other HIF-1α-dependent genes. Accordingly, macrophages lacking PKM2 also showed reduced expression of the HIF-1α-responsive genes *Il1*β and *Ldha* in response to LPS (23). Moreover, it was also demonstrated that PKM2 functions as a regulator of high mobility group box-1 (HMGB1) release by activated macrophages through interaction and activation of HIF-1α (58). HMGB1 is a ubiquitous nuclear protein that can be released by activated macrophages and act as a potent proinflammatory cytokine (60). The knockdown or inhibition of PKM2 using shRNA or shikonin, respectively, markedly reduces the release of HMGB1 by activated macrophages (58). Additionally, activation of colorectal carcinoma cells with LPS results in an increased production of TNF-α and IL-1β in a PKM2/STAT3-dependent manner. Mechanistically, LPS induces PKM2 nuclear translocation and binding to the STAT3 promoter, enhancing its transcription and subsequent activation (61). A recent report has also directly implicated a critical role for dimeric PKM2 in the hyper-inflammatory behavior of macrophages from coronary artery disease (CAD) patients (59). It was shown that nuclear translocation of dimeric PKM2 results in phosphorylation of STAT3 in LPS-activated CAD macrophages, boosting IL-1β and IL-6 transcription. Forcing PKM2 into tetramer conformation with ML265 prevented its LPS-induced nuclear translocation and STAT3 phosphorylation. Thus, PKM2 seems to be a critical regulator of expression and secretion of proinflammatory mediators, highlighting the possibility of targeting this protein in the treatment of inflammatory and infectious diseases.

Indeed, inhibition of dimeric PKM2 by shikonin conferred significant protection of mice against LPS-induced endotoxemia (58). Furthermore, mice treated with TEPP-46 showed reduced produced of IL-1β in response to LPS and *Salmonella typhimurium*-induced production *in vivo* (23). In line with these observations, studies in recent years have reported increased expression of PKM2 in different inflammatory disorders. The expression of PKM2 in intestinal tissue was found at high levels in patients with Crohn’s disease and positively correlated with disease activity scores or serum inflammatory markers (62). Moreover, elevated levels of PKM2 were found in stool samples from patients with active Crohn’s disease, suggesting that this protein can be a useful non-invasive marker for inflammatory bowel disease (63, 64). In accordance with this, expression of PKM2 was progressively increased in intestinal tissue of mice undergoing TNBS-induced colitis (62, 65). Finally, proteomic analysis revealed that PKM2 was one of the 33 over-expressed proteins found in synovial tissue from patients with rheumatoid arthritis (66). These findings indicate that PKM2 expression is upregulated in a multitude of inflammatory disorders. However, further studies are warranted to understand the regulatory functions of PKM2 on different inflammatory conditions.

## Perspectives and Conclusion

During the past years, metabolism and immunology have existed as two distinct fields of investigation, but there is now a general consensus that they intersect at several points. The concept of metabolic reprograming as a mechanism to drive an inflammatory response has mainly focused on how an immune cell’s metabolic status can directly influence its activity and function. In recent years, PKM2 has emerged not just as a key regulator of metabolic reprograming but also as a key player in controlling the transcription of critical genes in cancer cells, and most recently, in immune cells.

The current strategy for the treatment of inflammatory diseases is fundamentally based on interrupting the production or action of mediators that orchestrate the host’s response to tissue injury. An ideal drug to treat inflammatory disease would be able to both turn off the inflammatory response as well as activate the resolution program, including the induction of neutrophil apoptosis and polarization of macrophages into M2 (alternatively activated or pro-resolution) phenotype. Notably, recent studies show that PKM2 regulates the expression of proinflammatory mediators, prevents apoptosis, and drives macrophage polarization toward M1 phenotype (23, 58, 61, 67–69), indicating the potential of this enzyme as a target for the development of anti-inflammatory and proresolutive therapies.

Furthermore, recent studies have unraveled a notable involvement of PKM2 in controlling the transcriptional activity of HIF-1α and STAT3 pathways during inflammation. The expression and enzymatic activity of PKM2 can be regulated at multiple levels, including transcription, posttranslational modifications, and allosteric regulation of conformational stability. Therefore, PKM2 represents a novel potential target for the development of anti-inflammatory drugs.

## Author Contributions

All authors listed have made substantial, direct, and intellectual contribution to the work, and approved it for publication.

## Conflict of Interest Statement

The authors declare that the research was conducted in the absence of any commercial or financial relationships that could be construed as a potential conflict of interest.
